# Bis­(guanidinium) naphthalene-1,5-di­sulfonate–18-crown-6 (1/1)

**DOI:** 10.1107/S1600536812009154

**Published:** 2012-03-07

**Authors:** Bin Wei

**Affiliations:** a Ordered Matter Science Research Center, Southeast University, Nanjing 211189, People’s Republic of China

## Abstract

In the crystal of the title compound, 2CH_6_N_3_^+^·C_10_H_6_O_6_S_2_^2−^·C_12_H_24_O_6_, the 1,5-naphthnalenedisulfonate anion and the 18-crown-6 mol­ecule lie across inversion centers. The guanidin­ium cation links with the 1,5-naphthnalenedisulfonate anion and 18-crown-6 mol­ecule *via* N—H⋯O hydrogen bonds.

## Related literature

For applications of crown ethers, see: Clark *et al.* (1998 ▶). The title compound was obtained during a search for new hydrogen-bonding-type dielectric materials. For ferroelectric metal-organic 18-crown-6 clathrates, see: Fu *et al.* (2009 ▶, 2011 ▶); Ye *et al.* (2006 ▶); Zhang *et al.* (2008 ▶, 2010 ▶).
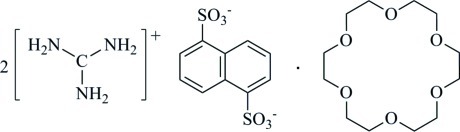


## Experimental

### 

#### Crystal data

2CH_6_N_3_^+^·C_10_H_6_O_6_S_2_^−^·C_12_H_24_O_6_
*M* *_r_* = 670.76
Triclinic, 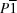

*a* = 8.5275 (17) Å
*b* = 9.1291 (18) Å
*c* = 11.470 (2) Å
α = 111.97 (3)°
β = 96.10 (3)°
γ = 99.38 (3)°
*V* = 803.3 (3) Å^3^
*Z* = 1
Mo *K*α radiation
μ = 0.23 mm^−1^
*T* = 293 K
0.20 × 0.20 × 0.20 mm

#### Data collection

Rigaku SCXmini diffractometer
8245 measured reflections
3638 independent reflections
3070 reflections with *I* > 2σ(*I*)
*R*_int_ = 0.025

#### Refinement

*R*[*F* ^2^ > 2σ(*F* ^2^)] = 0.043
*wR*(*F* ^2^) = 0.104
*S* = 1.05
3638 reflections
223 parameters
H atoms treated by a mixture of independent and constrained refinement
Δρ_max_ = 0.34 e Å^−3^
Δρ_min_ = −0.46 e Å^−3^

### 

Data collection: *CrystalClear* (Rigaku, 2005 ▶); cell refinement: *CrystalClear*; data reduction: *CrystalClear*; program(s) used to solve structure: *SHELXTL* (Sheldrick, 2008 ▶); program(s) used to refine structure: *SHELXTL*; molecular graphics: *SHELXTL*; software used to prepare material for publication: *SHELXTL*.

## Supplementary Material

Crystal structure: contains datablock(s) I, Global. DOI: 10.1107/S1600536812009154/xu5475sup1.cif

Structure factors: contains datablock(s) I. DOI: 10.1107/S1600536812009154/xu5475Isup2.hkl

Supplementary material file. DOI: 10.1107/S1600536812009154/xu5475Isup3.cml

Additional supplementary materials:  crystallographic information; 3D view; checkCIF report

## Figures and Tables

**Table 1:** Hydrogen-bond geometry (Å, °)

*D*—H⋯*A*	*D*—H	H⋯*A*	*D*⋯*A*	*D*—H⋯*A*
N1—H24⋯O2^i^	0.75 (3)	2.36 (3)	2.897 (3)	131 (2)
N1—H25⋯O2^ii^	0.84 (3)	2.05 (3)	2.887 (3)	175 (3)
N2—H22⋯O6^iii^	0.81 (3)	2.28 (3)	3.029 (3)	154 (3)
N2—H23⋯O5^iii^	0.80 (3)	2.43 (3)	2.867 (3)	115 (3)
N3—H20⋯O3^ii^	0.90 (3)	2.01 (3)	2.913 (3)	179 (2)
N3—H21⋯O4	0.81 (3)	2.18 (3)	2.952 (3)	159 (2)

Symmetry codes: (i) 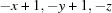; (ii) 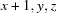; (iii) 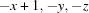.

**Fig. 1.**
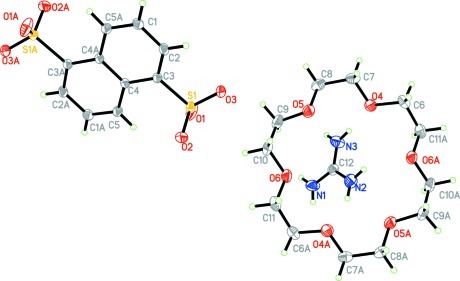
The molecular structure of the title compound, with the atomic numbering scheme. Displacement ellipsoids are drawn at the 30% probability level.

**Fig. 2.**
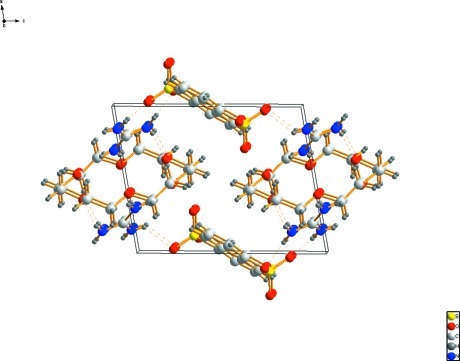
A view of the packing of the title compound, stacking along the b axis. Dashed lines indicate hydrogen bonds.

## supplementary crystallographic information

### Comment

Recent years, crown ethers have attracted much attention because of their wide application in catalysis, solvent extraction, isotopeseparation, bionice, host–guest chemistry and supramolecular chemistry (Clark *et al.*, 1998). Several 18-crown-6 clathrates were discovered to be dielectric-ferroelectric materials (Fu *et al.*, 2011), hence we design the title compound to find new hydrogen bonding type dielectric materials. Dielectric-ferroelectric materials, comprising organic ligands, metal-organic coordination compounds and organic-inorganic hybrids almost show dielectric constant of temperature-dependent (Fu *et al.*, 2009; Zhang *et al.*, 2010; Zhang *et al.*, 2008; Ye *et al.*, 2006). Unfortunately, the dielectric constant of the title compound as a function of temperature indicates that the permittivity is basically temperature-independent, below the melting point (395k-396k) of the compound, we have found that title compound has no dielectric disuniform from 80 K to 405 K. Herein we descibe the crystal structure of this compound.

At home temperature (25°C), the single-crystal X-ray diffraction reveals that the structure get crystallization in the triclinic system, space group P-1 and the asymmetric unit of the title compound consists of a guanidinium cation, a 1,5-naphthalenedisulfonate anion and a 18-crown-6 molecule (Fig. 1). The three –NH_2_^+^ groups form guanidinium interact with three O atoms of one crown ether molecule and other three O atoms from two 1,5-naphthalenedisulfonate anions through six N—H···O hydraogen bonds (Table 1), composing a three-dimensional crystal structure (Fig. 2).

### Experimental

The 1,5-naphthalene disulfonic acid (1.15 g, 4 mmol) and guanidinium tetrafluoroborate (1.17 g, 8 mmol) were dissolved in 30 ml water and the solution was combined with methanol solution of dibenzo-18-crown-6 (1.44 g 4 mmol). The mixture solution was stirred for 30 min to reaction fully and good quality blocky single crystals were obtained by slow evaporation of the filtrate after two weeks (the chemical yield 61%).

### Refinement

Amino H atoms were located in a difference Fourier map and refined isotropically. Other H atoms were placed in geometrically idealized positions nd constrained to ride on their parent atoms with C—H = 0.93–0.97 Å, *U*_iso_(H) = 1.2*U*_iso_(C).

### Crystal data

**Table:**

2CH_6_N_3_^+^·C_10_H_6_O_6_S_2_^−^·C_12_H_24_O_6_	*Z* = 1
*M**_r_* = 670.76	*F*(000) = 356
Triclinic, *P*1	*D*_x_ = 1.387 Mg m^−^^3^
Hall symbol: -P 1	Mo *K*α radiation, λ = 0.71073 Å
*a* = 8.5275 (17) Å	Cell parameters from 3638 reflections
*b* = 9.1291 (18) Å	θ = 3.0–27.5°
*c* = 11.470 (2) Å	µ = 0.23 mm^−^^1^
α = 111.97 (3)°	*T* = 293 K
β = 96.10 (3)°	Block, colorless
γ = 99.38 (3)°	0.20 × 0.20 × 0.20 mm
*V* = 803.3 (3) Å^3^	

### Data collection

**Table:**

Rigaku SCXmini diffractometer	3070 reflections with *I* > 2σ(*I*)
Radiation source: fine-focus sealed tube	*R*_int_ = 0.025
Graphite monochromator	θ_max_ = 27.4°, θ_min_ = 3.1°
CCD_Profile_fitting scans	*h* = −11→10
8245 measured reflections	*k* = −11→11
3638 independent reflections	*l* = −14→14

### Refinement

**Table:**

Refinement on *F*^2^	Primary atom site location: structure-invariant direct methods
Least-squares matrix: full	Secondary atom site location: difference Fourier map
*R*[*F*^2^ > 2σ(*F*^2^)] = 0.043	Hydrogen site location: inferred from neighbouring sites
*wR*(*F*^2^) = 0.104	H atoms treated by a mixture of independent and constrained refinement
*S* = 1.05	*w* = 1/[σ^2^(*F*_o_^2^) + (0.0379*P*)^2^ + 0.3633*P*] where *P* = (*F*_o_^2^ + 2*F*_c_^2^)/3
3638 reflections	(Δ/σ)_max_ < 0.001
223 parameters	Δρ_max_ = 0.34 e Å^−^^3^
0 restraints	Δρ_min_ = −0.46 e Å^−^^3^

### Special details

**Table:**

Geometry. All e.s.d.'s (except the e.s.d. in the dihedral angle between two l.s. planes) are estimated using the full covariance matrix. The cell e.s.d.'s are taken into account individually in the estimation of e.s.d.'s in distances, angles and torsion angles; correlations between e.s.d.'s in cell parameters are only used when they are defined by crystal symmetry. An approximate (isotropic) treatment of cell e.s.d.'s is used for estimating e.s.d.'s involving l.s. planes.
Refinement. Refinement of *F*^2^ against ALL reflections. The weighted *R*-factor *wR* and goodness of fit *S* are based on *F*^2^, conventional *R*-factors *R* are based on *F*, with *F* set to zero for negative *F*^2^. The threshold expression of *F*^2^ > σ(*F*^2^) is used only for calculating *R*-factors(gt) *etc*. and is not relevant to the choice of reflections for refinement. *R*-factors based on *F*^2^ are statistically about twice as large as those based on *F*, and *R*- factors based on ALL data will be even larger.

### Fractional atomic coordinates and isotropic or equivalent isotropic displacement parameters (Å^2^)

**Table:**

	*x*	*y*	*z*	*U*_iso_*/*U*_eq_	
S1	0.11339 (5)	0.65053 (5)	0.31289 (4)	0.03167 (13)	
O3	0.09429 (17)	0.50066 (14)	0.33254 (12)	0.0414 (3)	
O4	0.63864 (17)	−0.01790 (16)	0.23799 (13)	0.0459 (3)	
O5	0.47238 (18)	0.22644 (16)	0.24927 (13)	0.0490 (4)	
C3	0.02394 (19)	0.78364 (18)	0.43259 (15)	0.0277 (3)	
O6	0.40620 (17)	0.26914 (16)	0.01217 (13)	0.0468 (3)	
C4	0.03593 (18)	0.94983 (18)	0.45050 (15)	0.0263 (3)	
C1	−0.1274 (2)	0.8214 (2)	0.60430 (18)	0.0387 (4)	
H1	−0.1821	0.7782	0.6541	0.046*	
O1	0.28092 (17)	0.73046 (16)	0.33171 (17)	0.0608 (5)	
C2	−0.0571 (2)	0.7217 (2)	0.50692 (17)	0.0345 (4)	
H2	−0.0658	0.6134	0.4931	0.041*	
O2	0.0209 (2)	0.62768 (18)	0.19082 (13)	0.0574 (4)	
C12	0.7991 (2)	0.1911 (2)	0.04975 (18)	0.0415 (4)	
N3	0.8556 (3)	0.2086 (3)	0.16648 (19)	0.0569 (5)	
C5	0.1159 (2)	1.0194 (2)	0.37412 (17)	0.0349 (4)	
H5	0.1611	0.9550	0.3085	0.042*	
N2	0.6973 (3)	0.0547 (2)	−0.0292 (2)	0.0626 (6)	
C8	0.5725 (3)	0.2333 (3)	0.35990 (19)	0.0539 (5)	
H8A	0.5319	0.2912	0.4361	0.065*	
H8B	0.6820	0.2903	0.3672	0.065*	
C11	0.3022 (3)	0.2636 (3)	−0.0967 (2)	0.0532 (5)	
H11A	0.2955	0.3727	−0.0865	0.064*	
H11B	0.1943	0.2037	−0.1034	0.064*	
C9	0.4799 (3)	0.3797 (2)	0.2418 (2)	0.0575 (6)	
H9A	0.5878	0.4217	0.2326	0.069*	
H9B	0.4563	0.4558	0.3197	0.069*	
C7	0.5717 (3)	0.0633 (3)	0.34750 (19)	0.0493 (5)	
H7A	0.6351	0.0655	0.4238	0.059*	
H7B	0.4619	0.0058	0.3382	0.059*	
C6	0.6336 (3)	−0.1836 (3)	0.2156 (2)	0.0501 (5)	
H6A	0.5231	−0.2393	0.2066	0.060*	
H6B	0.6981	−0.1898	0.2879	0.060*	
N1	0.8482 (3)	0.3087 (3)	0.0127 (2)	0.0653 (6)	
C10	0.3593 (3)	0.3611 (3)	0.1293 (2)	0.0552 (6)	
H10A	0.2537	0.3070	0.1334	0.066*	
H10B	0.3520	0.4672	0.1318	0.066*	
H20	0.928 (3)	0.299 (3)	0.219 (2)	0.066 (7)*	
H21	0.819 (3)	0.142 (3)	0.193 (2)	0.065 (8)*	
H25	0.903 (3)	0.399 (3)	0.065 (3)	0.074 (9)*	
H22	0.670 (3)	−0.012 (3)	0.000 (3)	0.070 (9)*	
H24	0.829 (3)	0.289 (3)	−0.057 (3)	0.059 (8)*	
H23	0.659 (4)	0.047 (4)	−0.099 (3)	0.094 (11)*	

### Atomic displacement parameters (Å^2^)

**Table:**

	*U*^11^	*U*^22^	*U*^33^	*U*^12^	*U*^13^	*U*^23^
S1	0.0417 (2)	0.0225 (2)	0.0323 (2)	0.00950 (16)	0.01195 (17)	0.01017 (16)
O3	0.0617 (9)	0.0267 (6)	0.0411 (7)	0.0161 (6)	0.0153 (6)	0.0150 (5)
O4	0.0520 (8)	0.0399 (7)	0.0458 (8)	0.0041 (6)	0.0122 (6)	0.0188 (6)
O5	0.0553 (9)	0.0386 (7)	0.0412 (7)	0.0067 (6)	−0.0003 (6)	0.0068 (6)
C3	0.0293 (8)	0.0254 (8)	0.0291 (8)	0.0073 (6)	0.0054 (6)	0.0108 (6)
O6	0.0498 (8)	0.0426 (7)	0.0513 (8)	0.0232 (6)	0.0100 (6)	0.0168 (6)
C4	0.0264 (8)	0.0250 (7)	0.0294 (8)	0.0065 (6)	0.0055 (6)	0.0126 (6)
C1	0.0454 (10)	0.0369 (9)	0.0487 (11)	0.0129 (8)	0.0237 (9)	0.0279 (8)
O1	0.0477 (8)	0.0351 (7)	0.0928 (12)	0.0100 (6)	0.0374 (8)	0.0111 (7)
C2	0.0409 (9)	0.0260 (8)	0.0425 (10)	0.0091 (7)	0.0125 (8)	0.0182 (7)
O2	0.0952 (12)	0.0506 (8)	0.0305 (7)	0.0300 (8)	0.0092 (7)	0.0157 (6)
C12	0.0424 (10)	0.0352 (9)	0.0421 (10)	0.0033 (8)	0.0097 (8)	0.0118 (8)
N3	0.0583 (12)	0.0525 (11)	0.0517 (11)	−0.0154 (9)	−0.0037 (9)	0.0265 (10)
C5	0.0416 (10)	0.0326 (9)	0.0391 (9)	0.0140 (7)	0.0197 (8)	0.0182 (7)
N2	0.0723 (14)	0.0400 (11)	0.0573 (13)	−0.0042 (10)	−0.0098 (11)	0.0118 (10)
C8	0.0543 (13)	0.0537 (13)	0.0358 (11)	0.0051 (10)	0.0011 (9)	0.0027 (9)
C11	0.0518 (12)	0.0508 (12)	0.0676 (14)	0.0241 (10)	0.0093 (11)	0.0305 (11)
C9	0.0706 (15)	0.0356 (10)	0.0540 (13)	0.0146 (10)	0.0116 (11)	0.0031 (9)
C7	0.0464 (11)	0.0597 (13)	0.0354 (10)	0.0030 (10)	0.0027 (9)	0.0169 (9)
C6	0.0518 (12)	0.0495 (12)	0.0577 (13)	0.0097 (9)	0.0050 (10)	0.0328 (10)
N1	0.0971 (18)	0.0497 (12)	0.0444 (12)	−0.0041 (11)	0.0146 (12)	0.0215 (10)
C10	0.0662 (14)	0.0402 (11)	0.0608 (14)	0.0278 (10)	0.0200 (11)	0.0133 (10)

### Geometric parameters (Å, º)

**Table:**

S1—O1	1.4481 (15)	C5—C1^i^	1.365 (2)
S1—O3	1.4544 (12)	C5—H5	0.9300
S1—O2	1.4556 (15)	N2—H22	0.81 (3)
S1—C3	1.7943 (17)	N2—H23	0.80 (3)
O4—C6	1.429 (2)	C8—C7	1.504 (3)
O4—C7	1.430 (2)	C8—H8A	0.9700
O5—C9	1.425 (3)	C8—H8B	0.9700
O5—C8	1.427 (2)	C11—C6^ii^	1.496 (3)
C3—C2	1.372 (2)	C11—H11A	0.9700
C3—C4	1.437 (2)	C11—H11B	0.9700
O6—C10	1.431 (2)	C9—C10	1.499 (3)
O6—C11	1.432 (2)	C9—H9A	0.9700
C4—C5	1.428 (2)	C9—H9B	0.9700
C4—C4^i^	1.434 (3)	C7—H7A	0.9700
C1—C5^i^	1.365 (2)	C7—H7B	0.9700
C1—C2	1.412 (2)	C6—C11^ii^	1.496 (3)
C1—H1	0.9300	C6—H6A	0.9700
C2—H2	0.9300	C6—H6B	0.9700
C12—N3	1.314 (3)	N1—H25	0.84 (3)
C12—N1	1.319 (3)	N1—H24	0.75 (3)
C12—N2	1.327 (3)	C10—H10A	0.9700
N3—H20	0.90 (3)	C10—H10B	0.9700
N3—H21	0.81 (3)		
			
O1—S1—O3	113.09 (9)	O5—C8—H8B	110.0
O1—S1—O2	112.57 (11)	C7—C8—H8B	110.0
O3—S1—O2	111.83 (9)	H8A—C8—H8B	108.3
O1—S1—C3	107.06 (9)	O6—C11—C6^ii^	109.94 (16)
O3—S1—C3	106.12 (8)	O6—C11—H11A	109.7
O2—S1—C3	105.53 (8)	C6^ii^—C11—H11A	109.7
C6—O4—C7	112.50 (15)	O6—C11—H11B	109.7
C9—O5—C8	113.94 (16)	C6^ii^—C11—H11B	109.7
C2—C3—C4	120.72 (15)	H11A—C11—H11B	108.2
C2—C3—S1	117.84 (12)	O5—C9—C10	109.51 (17)
C4—C3—S1	121.44 (12)	O5—C9—H9A	109.8
C10—O6—C11	111.63 (15)	C10—C9—H9A	109.8
C5—C4—C4^i^	118.55 (17)	O5—C9—H9B	109.8
C5—C4—C3	122.97 (14)	C10—C9—H9B	109.8
C4^i^—C4—C3	118.48 (17)	H9A—C9—H9B	108.2
C5^i^—C1—C2	120.57 (15)	O4—C7—C8	109.21 (17)
C5^i^—C1—H1	119.7	O4—C7—H7A	109.8
C2—C1—H1	119.7	C8—C7—H7A	109.8
C3—C2—C1	120.44 (15)	O4—C7—H7B	109.8
C3—C2—H2	119.8	C8—C7—H7B	109.8
C1—C2—H2	119.8	H7A—C7—H7B	108.3
N3—C12—N1	119.0 (2)	O4—C6—C11^ii^	109.89 (17)
N3—C12—N2	119.6 (2)	O4—C6—H6A	109.7
N1—C12—N2	121.4 (2)	C11^ii^—C6—H6A	109.7
C12—N3—H20	119.7 (16)	O4—C6—H6B	109.7
C12—N3—H21	119.9 (18)	C11^ii^—C6—H6B	109.7
H20—N3—H21	120 (2)	H6A—C6—H6B	108.2
C1^i^—C5—C4	121.22 (16)	C12—N1—H25	120.7 (19)
C1^i^—C5—H5	119.4	C12—N1—H24	116 (2)
C4—C5—H5	119.4	H25—N1—H24	123 (3)
C12—N2—H22	116.7 (19)	O6—C10—C9	110.54 (17)
C12—N2—H23	119 (2)	O6—C10—H10A	109.5
H22—N2—H23	124 (3)	C9—C10—H10A	109.5
O5—C8—C7	108.65 (16)	O6—C10—H10B	109.5
O5—C8—H8A	110.0	C9—C10—H10B	109.5
C7—C8—H8A	110.0	H10A—C10—H10B	108.1

Symmetry codes: (i) −*x*, −*y*+2, −*z*+1; (ii) −*x*+1, −*y*, −*z*.

### Hydrogen-bond geometry (Å, º)

**Table:**

*D*—H···*A*	*D*—H	H···*A*	*D*···*A*	*D*—H···*A*
N1—H24···O2^iii^	0.75 (3)	2.36 (3)	2.897 (3)	131 (2)
N1—H25···O2^iv^	0.84 (3)	2.05 (3)	2.887 (3)	175 (3)
N2—H22···O6^ii^	0.81 (3)	2.28 (3)	3.029 (3)	154 (3)
N2—H23···O5^ii^	0.80 (3)	2.43 (3)	2.867 (3)	115 (3)
N3—H20···O3^iv^	0.90 (3)	2.01 (3)	2.913 (3)	179 (2)
N3—H21···O4	0.81 (3)	2.18 (3)	2.952 (3)	159 (2)

Symmetry codes: (ii) −*x*+1, −*y*, −*z*; (iii) −*x*+1, −*y*+1, −*z*; (iv) *x*+1, *y*, *z*.
